# Maternal Restricted- and Over-Feeding During Gestation Result in Distinct Lipid and Amino Acid Metabolite Profiles in the Longissimus Muscle of the Offspring

**DOI:** 10.3389/fphys.2019.00515

**Published:** 2019-05-01

**Authors:** Dominique E. Martin, Amanda K. Jones, Sambhu M. Pillai, Maria L. Hoffman, Katelyn K. McFadden, Steven A. Zinn, Kristen E. Govoni, Sarah A. Reed

**Affiliations:** ^1^Department of Animal Science, University of Connecticut, Storrs, CT, United States; ^2^Department of Psychology, Providence College, Providence, RI, United States; ^3^Department of Pediatrics, School of Medicine, University of Colorado, Aurora, Aurora, CO, United States; ^4^School of Medicine, Georgetown University, Washington, DC, United States; ^5^Department of Fisheries, Animal and Veterinary Sciences, The University of Rhode Island, Kingston, RI, United States; ^6^Cummings School of Veterinary Medicine, Tufts University, North Grafton, MA, United States

**Keywords:** gestation, maternal diet, metabolism, muscle, offspring

## Abstract

Maternal over- and restricted-feeding during gestation have similar negative consequences for the offspring, including decreased muscularity, increased adiposity, and altered metabolism. Our objective was to determine the effects of poor maternal nutrition during gestation (over- and restricted-feeding) on the offspring muscle metabolite profile. Pregnant ewes (*n* = 47) were fed 60% (RES), 100% (CON), or 140% (OVER) of NRC requirements starting at day 30.2 ± 0.2 of gestation. Offspring sample collection occurred at days 90 and 135 of gestation, and within 24 h of birth. C2C12 myoblasts were cultured in serum collected from offspring at birth (*n* = 18; 6 offspring per treatment) for analysis of oxidative and glycolytic capacity. Unbiased metabolite analysis of longissimus muscle samples (*n* = 72; 8 fetuses per treatment per time point) was performed using mass spectrometry. Data were analyzed by ANOVA for main effects of treatment, time point, and their interaction. Cells cultured in serum from RES offspring exhibited increased proton leak 49% (*p* = 0.01) compared with CON, but no other variables of mitochondrial respiration or glycolytic function were altered. Mass spectrometry identified 612 metabolites. Principle component analysis identified day of gestation as the primary driver of metabolic change; however, maternal diet also altered the lipid and amino acid profiles in offspring. The abundance of 53 amino acid metabolites and 89 lipid metabolites was altered in RES compared with CON (*p* ≤ 0.05), including phospholipids, sphingolipids, and ceramides within the lipid metabolism pathway and metabolites involved in glutamate, histidine, and glutathione metabolism. Similarly, abundance of 63 amino acid metabolites and 70 lipid metabolites was altered in OVER compared with CON (*p* ≤ 0.05). These include metabolites involved in glutamate, histidine, lysine, and tryptophan metabolism and phosphatidylethanolamine, lysophospholipids, and fatty acids involved in lipid metabolism. Further, the amino acid and lipid profiles diverged between RES and OVER, with 69 amino acid and 118 lipid metabolites differing (*p* ≤ 0.05) between groups. Therefore, maternal diet affects metabolite abundance in offspring longissimus muscle, specifically metabolites involved in lipid and amino metabolism. These changes may impact post-natal skeletal muscle metabolism, possibly altering energy efficiency and long-term health.

## Introduction

Poor maternal nutrition during gestation can negatively impact offspring growth and development pre- and post-natally. Both restricted- and over-feeding during gestation can lead to alterations in body size and birth weight of offspring, as well as impaired muscle growth, changes in adiposity, and altered metabolism in multiple species which have long-lasting and detrimental effects on offspring growth and health (Hales and Barker, 2001; Wu et al., 2006; Reed et al., 2014; Hoffman et al., 2017). These long-lasting post-natal effects can persist into adulthood, and are likely the result of fetal programming, which is defined as changes in the maternal environment that alter the pre-natal development and post-natal growth trajectory of the developing offspring (Nathanielsz et al., 2007). Additionally, the impacts of poor maternal nutrition on offspring metabolism are linked with increased incidence of metabolic dysregulation and disease in the adult (Hales and Ozanne, 2003).

Because fiber number is determined during fetal development, muscle tissue is particularly vulnerable to intrauterine conditions, such as changes in maternal nutrition. Although there is an increase in net muscle mass after birth due to muscle fiber hypertrophy, the number of muscle fibers is set during gestation and no fiber hyperplasia occurs post-natally (Rowe and Goldspink, 1969; Beermann et al., 1978; Wigmore and Stickland, 1983; White et al., 2010). Currently, knowledge of the effects of maternal diet on muscle are primarily focused on phenotypic changes. For example, restricted-feeding dams during gestation increased offspring muscle fiber cross-sectional area (CSA) of semitendinosus muscle fibers at birth but resulted in reduced post-natal growth and smaller muscle fiber CSA at 3 months of age (Reed et al., 2014). Further, 8-mo-old lambs born to nutrient deficient mothers had fewer total muscle fibers than control animals (Zhu et al., 2006). Maternal over-feeding also increased muscle and whole-body adiposity in the offspring during fetal development, at birth, and post-natally (Daniel et al., 2007; Tong et al., 2009; Reed et al., 2014). In addition, maternal over-feeding during gestation decreased the number of muscle fibers formed during development and the size of those fibers pre-natally and post-natally (Cerisuelo et al., 2009; Tong et al., 2009; Reed et al., 2014). Muscle has an integral role in metabolism and inter-organ crosstalk due to its involvement with glucose and protein utilization and is susceptible to fetal programming in response to poor maternal nutrition. Skeletal muscle comprises 40–50% of body mass, making it the most abundant insulin-sensitive tissue (DeFronzo et al., 1985; Baron et al., 1988; Zurlo et al., 1990). Further, skeletal muscle is responsible for 75–95% of insulin-mediated glucose disposal and 20–30% of whole-body oxygen consumption (DeFronzo et al., 1985; Baron et al., 1988; Zurlo et al., 1990). Skeletal muscle also serves as an amino acid reservoir that sustains protein synthesis within the muscle and whole body (Wolfe, 2006). Changes to muscle mass, metabolic rate, hormone concentrations, or other circulating factors affecting muscle could significantly alter energy substrate use.

The altered uterine environment resulting from poor maternal nutrition during gestation may result in lasting changes to muscle metabolism and function, affecting post-natal muscle growth and whole-body metabolism. We hypothesized that poor maternal nutrition during gestation (over- and restricted-feeding) would alter the offspring muscle metabolome at mid- and late-gestation, and within 24 h of birth.

## Materials and Methods

### Animals

All animal procedures were reviewed and approved by the University of Connecticut Institutional Animal Care and Use Committee (A13-059). Full experimental details were previously reported in Pillai et al. (2017). Briefly, multiparous Western White-faced ewes (*n* = 47) were estrus synchronized using a controlled intravaginal drug release device (CIDR; Easi-Breed CIDR Sheep Insert, Zoetis, Parsippany, NJ, United States) and an intramuscular injection of PGF_2α_ [Lutalyse, 5 mg/mL; Zoetis, Inc.; (Knights et al., 2001)]. Ewes were bred to 1 of 4 related Dorset rams. Day 0 of pregnancy was determined when ewes received a rump mark and showed no further evidence of remarking. Twenty days later, ewes were moved to individual pens and transitioned over a 7-day period to a complete pelleted feed based on National Research Council (NRC) requirements for ewes gestating with twins. Ewes were confirmed pregnant using transabdominal ultrasound on day 28.5 ± 0.4 (Jones et al., 2016). Pregnant ewes (*n* = 5 to 7 per treatment per time point) were fed either a control- (100% NRC), restricted- (60% NRC), or over-fed diet (140% NRC) starting at day 30.2 ± 0.2 of gestation based on the NRC requirement for total digestible nutrients (TDN; National Research Council, 1985) and adjusted weekly for individual changes in body weight. Offspring from control-, restricted-, or over-fed ewes are denoted as CON, RES, and OVER, respectively. Diets effectively altered maternal body weight, with restricted-fed ewes having lighter body weights than control- and over-fed ewes as early as day 45 of gestation (Pillai et al., 2017). At birth, restricted-fed ewes weighed 12% less than control-fed ewes, and over-fed ewes were 11% heavier than control ewes (Pillai et al., 2017).

### Sample Collection

On days 90 and 135 of gestation, ewes (*n* = 5 to 7 per treatment per time point) were euthanized by an intravenous injection of Beuthanasia-D Special (Merck Animal Health; Summit, NJ, United States) containing 390 mg/mL sodium pentobarbital and 50 mg/mL phenytoin based on body weight, and exsanguinated. The fetuses were removed following a hysterectomy for necropsy and tissue collection. A subset of ewes was allowed to undergo parturition (birth; *n* = 5–7 per dietary treatment). Whole blood was obtained from live lambs via jugular venipuncture within 24 h of parturition and processed for serum (Hoffman et al., 2014). Subsequently, lambs were weighed and euthanized with an i.v. overdose of Beuthanasia-D Special (390 ng/mL sodium pentobarbital and 50 mg/mL phenytoin based on body weight), and exsanguinated. For the purposes of these experiments, a total of 72 offspring (*n* = 8 per treatment per time point) from 47 ewes at days 90 and 135, and within 24 h of birth were included in the final analysis. These time points represent mid-gestation, which is the time period of secondary myogenesis, late gestation, during which muscle growth is primarily through muscle hypertrophy, and just after parturition. Offspring body weight was not different at day 90 or day 135 of gestation, but RES offspring were lighter at birth than CON and OVER (*p* = 0.03; Pillai et al., 2017). Longissimus muscle (LM) samples from each offspring at days 90, 135, and birth were collected immediately after euthanasia and snap frozen in liquid nitrogen. The longissimus muscle is economically relevant as a dietary protein source. Samples were stored at -80°C until further use.

### C2C12 Cell Culture

C2C12 cells (ATCC CRL-1772, Manassas, VA, United States) were cultured in growth media containing Dulbecco’s modified Eagle media (DMEM) with 4 g/L D-glucose (Gibco Laboratories, Gaithersburg, MD, United States) and 10% fetal bovine serum (FBS), 1% penicillin/streptomycin, 0.3% fungizone, and 0.2% gentamycin. Cells were passed once and plated on 24-well plates at a density of 3,000 cells per well. After 24 h, the media was changed so that, instead of FBS, the growth media contained 10% offspring serum collected at birth from CON (*n* = 8), RES (*n* = 8), or OVER (*n* = 8) lambs. After 48 h, cells were pulsed with 5-bromo-2′-deoxyuridine (BrdU) for 30 min (Click-iT EdU Alexa Fluor 488 Imaging Kit, Invitrogen, Carlsbad, CA, United States), fixed with 4% paraformaldehyde, and immunostained according to manufacturer’s recommendations. Cells were incubated with Hoescht 33342 for 1 h to visualize nuclei. The cells were imaged using a Zeiss Observer, and 5 random images per well were taken. The percentage of proliferating cells was determined by the number of BrdU(+) cells divided by the total number of nuclei and multiplied by 100.

To analyze myoblast differentiation, C2C12 cells were cultured in growth media on 24-well plates. After reaching 100% confluency, growth media was replaced with differentiation media containing 2% offspring serum, 1% penicillin/streptomycin, 0.3% fungizone, and 0.2% gentamycin in DMEM with 1 g/L glucose. C2C12 cells were allowed to differentiate in treatment media for 5 days. Myotubes were fixed with 4% paraformaldehyde and blocked in 5% horse serum in phosphate buffered saline (PBS) and 0.1% triton-X. Cells were incubated with anti-Myosin heavy chain (MyHC; MF20, Developmental Hybridoma Core) overnight at 4°C. After extensive washing in PBS, cells were incubated with secondary antibody (Alexa Fluor 488; 1:500) for 1 h. Hoescht 33342 was used to visualize nuclei. The cells were imaged using a Zeiss Observer, and 5 random images per well were taken. Fusion index was calculated as the number of nuclei within multinucleated MyHC-positive myotubes divided by the number of total number of nuclei and multiplied by 100. Fiber diameter was measured in 3 places per myofiber and averaged per fiber.

### Mitochondrial and Glycolytic Stress Assays

Mitochondrial and glycolytic stress were evaluated using the Seahorse XFe24 Extracellular Flux Analyzer (Seahorse Bioscience; North Billerica, MA, United States) following manufacturer’s instructions and protocol. In brief, C2C12 cells were plated onto XFe24 culture plates at a density of 15,000 cells per well and cultured in 10% offspring serum in growth media [CON (*n* = 6), RES (*n* = 6), or OVER (*n* = 6)] for 24 h. Twelve hours before the assay, the XFe24 sensor cartridges were hydrated with Seahorse Bioscience XFe24 Calibrant (pH 7.4) and stored at 37°C.

For the Cell Mito Stress Test Assay, 1 mM pyruvate (Sigma-Aldrich, St. Louis, MO, United States), 2 mM glutamine (Sigma-Aldrich), and 10 mM glucose (Sigma-Aldrich) were added to the assay media, warmed to 37°C, pH was adjusted to 7.4 with 0.1 N NaOH, and the media was sterile filtered. Oligomycin, carbonyl cyanide-4 (trifluoromethoxy) phenylhydrazone (FCCP), and rotenone/antimycin A were prepared in assay media. The injection ports were loaded using the constant volume method described in the manufacturer’s protocol. The growth media was removed, and each well was rinsed twice with assay media. Assay media (525 μL) was added to each well, and the cell culture plate was incubated at 37°C without CO_2_ for 60 min. The plate was then loaded into the Seahorse XFe24 Extracellular Flux Analyzer and the standard operating procedure for Cell Mito Stress Test Assay for the machine was followed according to manufacturer’s protocol to measure oxygen consumption rate (OCR).

For the Glycolysis Stress Test Assay, 2 mM glutamine was added to Seahorse XF Assay Media (Seahorse Bioscience). The media was warmed to 37°C, pH was adjusted to 7.4 with 0.1 N NaOH, and the media was filter sterilized. The injection ports were loaded using the constant volume method described in the manufacturer’s protocol. The growth media was removed and each well was rinsed twice with assay media. Assay media (525 μL) was added to each well, and the cell culture plate was incubated at 37°C without CO_2_ for 60 min. The plate was then loaded into the Seahorse XFe24 Extracellular Flux Analyzer. Standard operating procedure for Glycolysis Stress Test Assay for the machine was followed according to manufacturer’s protocol to measure extracellular acidification rate (ECAR).

The DNA content in individual wells on the XFe24 cell culture plates was quantified to account for variations in cell density using Macherey-Nagel NucleoSpin Tissue kits (Macherey-Nagel Inc., Bethlehem, PA, United States), according to manufacturer’s protocol. The OCR and ECAR measurements were adjusted for total DNA content in each well.

### Metabolome Analysis

#### Sample Preparation

Longissimus dorsi samples (200 ng; *n* = 8 per treatment per time point) were shipped on dry ice to Metabolon, Inc. (Morrisville, NC, United States) for metabolome analysis. Samples were prepared using the automated MicroLab STAR^®^ system (Hamilton Company, Reno, NV, United States). Several recovery standards were added before the first step in the extraction process for quality control (QC) purposes. To remove protein, dissociate small molecules bound to protein or trapped in the precipitated protein matrix, and to recover chemically diverse metabolites, proteins were precipitated with methanol under vigorous shaking for 2 min (Glen Mills GenoGrinder 2000, SPEX Sample Prep, Metuchen, NJ, United States) followed by centrifugation. The resulting extract was divided into five fractions: two for analysis by two separate reverse phase (RP)/ultra-performance liquid chromatography-tandem mass spectrometry (UPLC-MS/MS) methods with positive ion mode electrospray ionization (ESI), one for analysis by RP/UPLC-MS/MS with negative ion mode ESI, one for analysis by Hydrophilic Interaction Chromatography (HILIC)/UPLC-MS/MS with negative ion mode ESI, and one sample was reserved for backup. Samples were placed briefly on a TurboVap (Zymark, Hopkinton, MA, United States) to remove the organic solvent. The sample extracts were stored overnight under nitrogen before preparation for analysis.

#### Ultrahigh Performance Liquid Chromatography-Tandem Mass Spectroscopy (UPLC-MS/MS)

All methods utilized a Waters ACQUITY UPLC and a Thermo Scientific Q-Exactive high resolution/accurate mass spectrometer interfaced with a heated electrospray ionization (HESI-II) source and Orbitrap mass analyzer operated at 35,000 mass resolution. The sample extract was dried, then reconstituted in solvents compatible with each of the four methods. Each reconstitution solvent contained a series of standards at fixed concentrations to ensure injection and chromatographic consistency. One aliquot was analyzed using acidic positive ion conditions, chromatographically optimized for more hydrophilic compounds. In this method, the extract was gradient eluted from a C18 column (Waters UPLC BEH C18-2.1 × 100 mm, 1.7 μm) using water and methanol, containing 0.05% perfluoropentanoic acid (PFPA) and 0.1% formic acid (FA). Another aliquot was also analyzed using acidic positive ion conditions; however, it was chromatographically optimized for more hydrophobic compounds. In this method, the extract was gradient eluted from the same afore mentioned C18 column using methanol, acetonitrile, water, 0.05% PFPA and 0.01% FA, and was operated at an overall greater organic content. Another aliquot was analyzed using basic negative ion optimized conditions using a separate dedicated C18 column. The basic extracts were gradient eluted from the column using methanol and water, however, with 6.5 mM Ammonium Bicarbonate at pH 8. The fourth aliquot was analyzed via negative ionization following elution from a HILIC column (Waters UPLC BEH Amide 2.1 × 150 mm, 1.7 μm) using a gradient consisting of water and acetonitrile with 10 mM Ammonium Formate, pH 10.8. Quality control was achieved using four separate controls: a pooled matrix sample generated by taking a small volume of each experimental sample served as a technical replicate throughout the data set; extracted water samples served as process blanks; and a cocktail of QC standards that were chosen not to interfere with the measurement of endogenous compounds were spiked into every analyzed sample allowed instrument performance monitoring and aided chromatographic alignment. Instrument variability was determined by calculating the median relative standard deviation (RSD) for the standards that were added to each sample prior to injection into the mass spectrometers. Overall process variability was determined by calculating the median RSD for all endogenous metabolites (i.e., non-instrument standards) present in 100% of the pooled matrix samples. Experimental samples were randomized across the platform run with QC samples spaced evenly among the injections. The mass spectrometer analysis alternated between mass spectrometer (MS) and data-dependent sequential mass spectrometry (MSn) scans using dynamic exclusion. The scan range varied slighted between methods but covered 70–1,000 m/z.

#### Data Extraction and Compound Identification

Raw data were extracted, peak-identified, and QC processed using Metabolon’s hardware and software. Compounds were identified by comparison with library entries of purified standards or recurrent unknown entities. Metabolon maintains a library based on authenticated standards that contains the retention time/index (RI), mass to charge ratio (m/z), and chromatographic data (including MS/MS spectral data) on all molecules present in the library. Furthermore, biochemical identifications are based on three criteria: retention index within a narrow RI window of the proposed identification, accurate mass match to the library ±10 ppm, and the MS/MS forward and reverse scores between the experimental data and authentic standards. The MS/MS scores are based on a comparison of the ions present in the experimental spectrum to the ions present in the library spectrum. While there may be similarities between these molecules based on one of these factors, the use of all three data points can be utilized to distinguish and differentiate compounds. More than 3,300 commercially available purified standard compounds have been acquired and registered into the laboratory information management system (LIMS) for analysis on all Metabolon platforms for determination of their analytical characteristics.

### Statistical Analysis

Cell culture data were analyzed using PROC MIXED (SAS Institute Inc., Cary, NC, United States; version 9.4). Main effects of treatment, sex, and their interaction were analyzed and differences between means were determined using the pdiff statement in LSMEANS. Data are reported as least square mean ± SE with significance at *p* ≤ 0.05.

Following log transformation and imputation of missing values with the minimum observed value for each compound, ANOVA contrasts were used to identify metabolites that differed significantly between diet, time and the interaction of diet and time. Statistical significance is declared for *p* ≤ 0.05 with false discovery rate *q* < 0.05. A principal component analysis was performed in Array Studio to find hierarchical clusterings of the data. Morpheus was used to generate heat maps using relative abundance (fold change) data^1^.

## Results

### Effects of RES and OVER Offspring Serum on Myoblast Characteristics

Serum from RES offspring increased proton leak 49% compared with CON (*p* = 0.01; Table 1), but serum from OVER offspring had no effect on proton leak compared with CON (*p* = 0.58). Serum from RES and OVER offspring did not significantly affect basal respiration, ATP production, maximal respiration, spare respiratory capacity, or non-mitochondrial respiration compared with CON (*p* ≥ 0.15). Serum from RES and OVER offspring did not significantly affect C2C12 glycolytic function (*p* ≥ 0.36; Table 2). There was no effect of sex (*p* ≥ 0.22) or interaction of treatment and sex (*p* ≥ 0.48) for any of the variables analyzed for mitochondrial or glycolytic function. Serum collected from RES and OVER offspring had no effect on BrdU incorporation (*p* ≥ 0.39; CON = 24.89 ± 3.03%, RES = 24.06 ± 3.03%, OVER = 27.74 ± 3.03%), myofiber fusion index (*p* ≥ 0.15; CON = 15.70 ± 2.87%, RES = 9.55 ± 3.23%, OVER = 10.97 ± 3.03%) or myofiber diameter (*p* ≥ 0.70; CON = 15.38 ± 1.33 μm, RES = 14.88 ± 1.46 μm, OVER = 15.25 ± 1.38 μm) compared with CON.

**Table 1:** Serum from offspring born to restricted-fed ewes increases C2C12 proton leak^1^.

	Maternal diet				
Variable^2^	CON	RES	OVER	SEM	*p*-value
Basal respiration	34.32	44.87	35.38	3.88	0.32
ATP production	19.33	22.54	18.94	3.21	0.69
Maximal respiration	177.15	303.21	182.58	49.83	0.17
Spare respiratory capacity	142.83	258.34	147.19	47.79	0.19
Proton leak	14.98^a^	22.33^b^	16.45^a^	1.81	0.01
Non-mitochondrial respiration	11.75	25.22	16.77	5.42	0.25

*^*1*^C2C12 cells were cultured in serum collected from offspring of control-fed (CON, n = 6), restricted (RES, n = 6), or over-fed (OVER, n = 6) ewes at birth. ^*2*^Measured as oxygen consumption rate (pmol O_2_ ⋅ min^-*1*^ ⋅ μg DNA^-*1*^). ^*a,b*^Values with different letters are significantly different, p <0.05.*

**Table 2:** Serum from offspring of poorly nourished ewes does not alter C2C12 glycolytic function^1^.

	Maternal diet				
Variable^2^	CON	RES	OVER	SEM	*p-*value
Glycolysis	48.03	45.21	41.39	4.08	0.27
Glycolytic capacity	51.41	52.35	47.85	4.75	0.51
Glycolytic reserve	3.38	7.14	6.46	1.91	0.19
Non-glycolytic acidification	15.63	16.69	14.33	1.93	0.40

*^*1*^C2C12 cells were cultured in serum collected from offspring of control-fed (CON, n = 6), restricted (RES, n = 6), or over-fed (OVER, n = 6) ewes at birth. ^*2*^Measured as extracellular acidification rate (mpH ⋅ min^-*1*^ ⋅ μg DNA^-*1*^).*

### Muscle Metabolome Analysis

**FIGURE 1:**
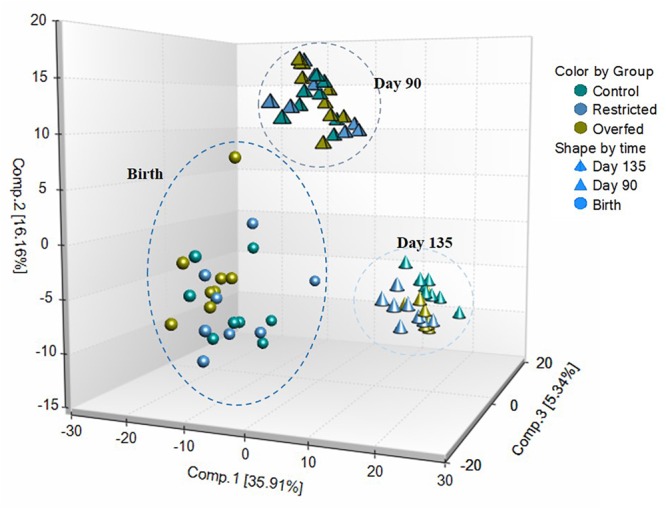
Principle component analysis (PCA) and hierarchical clustering obtained from employing metabolic concentration of all metabolites detected in longissimus muscle (LM). PCA of LM samples indicates that time is the primary driver of variance between samples. LM samples (*n* = 72; 8 per treatment per time point) were collected from offspring born to ewes fed control- (100%), restricted- (60%), or over-fed (140%) diets at days 90 or 135 of gestation, or within 24 h of birth were analyzed by UPLC-MS/MS. Offspring from control-, restricted-, or over-fed ewes are denoted as CON, RES, and OVER, respectively.

A total of 612 metabolites were identified in LM samples (Supplementary Table S1). Principle component analysis of LM samples (Figure 1) demonstrated clustering within day of gestation, indicating that gestational development is the primary driver of variance between samples. Accordingly, day of gestation altered metabolite abundance within each diet group (Table 3). However, maternal diet also altered metabolite abundance at each time point (Table 4). Maternal over- and restricted-feeding differentially altered metabolites in the 8 major metabolic pathways (amino acid, lipid, peptide, carbohydrate, energy, nucleotide, cofactors and vitamins, xenobiotics; Table 5). Maternal over-feeding altered 63 of 161 (39%) of identified metabolites in the amino acid metabolism pathways compared with CON, in contrast to RES offspring which demonstrated 53 of 161 (33%) metabolites altered compared with CON. Maternal restricted-feeding altered 89 of 268 (33%) identified metabolites in lipid metabolism compared with CON, in contrast to OVER offspring, in which 70 of 268 (26%) identified metabolites in lipid metabolism compared with controls. Because of the importance of amino acid metabolism in maintaining energy homeostasis in fetal offspring (Battaglia and Meschia, 1978), and transition to lipid metabolism during early post-natal life, we chose to focus on these classes of metabolites.

**Table 3:** Number of fetal metabolites in longissimus muscle altered by day of gestation.

		Metabolites^1^		
Diet^2^	Time	Total	Increased	Decreased
CON	day 90 vs. day 135	362	200	162
	day 90 vs. Birth	436	191	245
	day 135 vs. Birth	374	127	247
RES	day 90 vs. day 135	376	211	165
	day 90 vs. Birth	441	189	252
	day 135 vs. Birth	374	129	245
OVER	day 90 vs. day 135	374	219	155
	day 90 vs. Birth	441	230	211
	day 135 vs. Birth	365	160	205

*^*1*^Significance is determined at p ≤ 0.05. ^*2*^Offspring from ewes fed a control (100% NRC), restricted (60% NRC), or OVER (140% NRC) diet. Offspring from control-, restricted-, or over-fed ewes are denoted as CON, RES, and OVER, respectively.*

**Table 4:** Number of fetal metabolites in longissimus muscle altered by maternal diet.

		Metabolites^1^		
Diet^2^	Time	Total	Increased	Decreased
RES vs. CON	day 90	86	19	67
	day 135	72	22	50
	Birth	64	14	50
OVER vs. CON	day 90	78	49	29
	day 135	27	18	9
	Birth	102	15	87
RES vs. OVER	day 90	104	20	84
	day 135	118	38	80
	Birth	117	85	32

*^*1*^Significance is determined at p ≤ 0.05. ^*2*^Offspring from ewes fed a CON (100% NRC), RES (60% NRC), or OVER (140% NRC) diet. Offspring from control-, restricted-, or over-fed ewes are denoted as CON, RES, and OVER, respectively.*

**Table 5:** Number of metabolites in major pathways altered by maternal diet across time points^1^.

Major metabolic pathway	Total number of metabolites^2^	Pathway metabolites altered^3^		
		RES vs. CON	OVER vs. CON	RES vs. OVER
Amino acid	161	53 (33%)	63 (39%)	69 (43%)
Peptide	17	4 (24%)	6 (35%)	9 (53%)
Carbohydrate	39	8 (21%)	12 (31%)	9 (23%)
Energy	11	1 (9%)	1 (9%)	3 (27%)
Lipid	268	89 (33%)	70 (26%)	118 (44%)
Nucleotide	56	16 (29%)	10 (18%)	16 (29%)
Cofactors and vitamins	31	12 (39%)	15 (48%)	13 (42%)
Xenobiotics	29	13 (45%)	10 (34%)	14 (48%)

*^*1*^Offspring from ewes fed a CON (100% NRC), RES (60% NRC), or OVER (140% NRC) diet. Offspring from control-, restricted-, or over-fed ewes are denoted as CON, RES, and OVER, respectively. ^*2*^Number of metabolites identified within each pathway. ^*3*^Number of metabolites within each pathway altered between treatments, percent of total number of pathway metabolites is in parentheses. Significance is determined at p ≤ 0.05.*

#### Maternal Restricted-Feeding Alters Lipid Metabolism in the Offspring Longissimus Muscle

**FIGURE 2:**
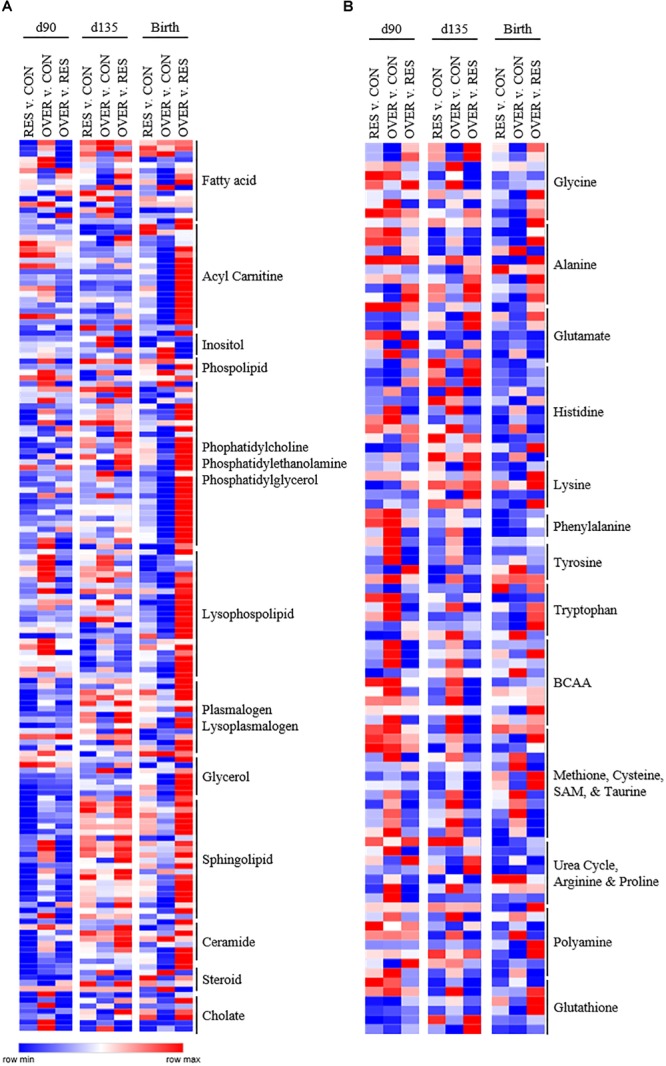
Poor maternal diet alters relative abundance of lipid **(A)** and amino acid **(B)** metabolites in the offspring longissimus muscle in a diet and time dependent manner. Offspring from ewes fed a control (CON; 100% NRC), over-fed (OVER; 140% NRC), or restricted-fed (RES; 60% NRC) diet were sampled at days 90 or 135 of gestation, or within 24 h of birth. Identified names are metabolite sub-pathways. Cells represent fold changes of abundance by color range, from red (row max) to white (row average) and blue (row minimum) between treatment groups.

Throughout gestation and immediately following parturition, RES demonstrated reduced abundance of phosphatidylcholines, phosphatidylethanolamines, and lysophospholipids compared with CON (*p* < 0.05; Figure 2). In particular, phosphatidyl-choline and phosphatidylethanolamine metabolites were decreased in RES compared with CON at day 90 of gestation (*p* < 0.05). Several lysophospholipid metabolites (Figure 2) were decreased in RES at each time point (*p* < 0.05), with the exception of 1-oleyl-glycerophosphoserine, which was increased at birth in RES compared with CON (*p* < 0.02). Acetyl carnitine and 3-hydroxybutyrylcarnitine were increased at birth, and 3-hydroxybutyrate was increased at day 135 in RES compared with CON (*p* < 0.04). Maternal restricted-feeding during gestation decreased abundance of 16 of 31 identified sphingomyelin metabolites at day 90 of gestation (*p* < 0.05; Figure 2). Abundance of two sphingolipid metabolites [sphingomyelin (d18:2/14:0, d18:1/14:1) and sphingomyelin (d18:2/24:2)] was increased in RES at day 135 compared with CON (*p* < 0.02). Coupled with changes in sphingomyelin metabolites were decreases in 7 of 10 identified ceramide metabolites at day 90; although abundance was similar to CON by day 135 of gestation.

#### Maternal Restricted-Feeding Alters Amino Acid Metabolism in the Offspring Longissimus Muscle

While the largest number of metabolites altered as a result of maternal restricted-feeding occurred in the lipid metabolism subpathways, maternal nutrient restriction also altered the abundance of metabolites in the amino acid metabolism subpathways (Figure 2). In the histidine metabolism subpathway, 1-methylhistidine, 3-methylhistidine, n-acetyl-1-methylhistidine, and anserine abundance was increased at day 135 relative to CON, while carnosine abundance was decreased (*p* < 0.03). At birth, abundance of carnosine, anserine, and 1-methyl-4-imidazoleacetate was decreased compared with CON (*p* < 0.05). Metabolites in the methionine, cysteine, SAM and taurine subpathway were also altered by restricted maternal nutrition. Specifically, *N*-acetylmethionine abundance was increased at day 90 of gestation (*p* = 0.03), while *N*-formylmethionine and taurocyamine abundance was decreased relative to CON at day 135 and birth (*p* < 0.02). Interestingly, reduced glutathione (GSH) was increased at day 90 (*p* = 0.01) with no change in oxidized glutathione (*p* = 0.74). 2-aminobutyrate was also decreased at day 90 of gestation but increased at day 135 of gestation relative to CON (*p* < 0.01).

#### Maternal Over-Feeding Alters Lipid Metabolism in the Offspring Longissimus

Maternal over-feeding during gestation altered the lipid metabolite profile in offspring muscle but affected fewer lipid metabolites than maternal restricted-feeding (Figure 2). In particular, four fatty acids [propionylcarnitine (C3); 5-dodecenoylcarnitine (C12:1); myristoleoylcarnitine (C14:1);behenoylcarnitine (C22)] were decreased in OVER at birth relative to CON (*p* < 0.02). Further, five phosphatidyl-ethanolamines [1-palmitoyl-2-oleoyl-GPE (16:0/18:1); 1-palmitoyl-2-arachidonoyl-GPE (16:0/20:4); 1-stearoyl-2-oleoyl-GPE (18:0/18:1); 1,2-dioleoyl-GPE (18:1/18:1); 1-oleoyl-2-linoleoyl-GPE (18:1/18:2)] and eight lysophospholipids [1-palmitoleoyl-GPC (16:1); 1-oleoyl-GPC (18:1); 1-linoleoyl-GPC (18:2); 1-palmitoyl-GPE (16:0); 1-oleoyl-GPE (18:1); 1-arachidonoyl-GPE (20:4n6); 1-palmitoyl-GPI (16:0); 1-stearoyl-GPI (18:0)] metabolites were decreased at birth in OVER (*p* < 0.05). No fatty acid, phosphatidylethanolamine, or lysophospholipids increased in OVER compared with CON at birth (*p* > 0.05).

#### Maternal Over-Feeding Alters Amino Acid Metabolism in the Offspring Longissimus Muscle

Increased maternal nutrient consumption during gestation altered amino acid metabolites in a variety of metabolic pathways (Figure 2). Of interest, 1-methylhistidine, 3-methylhistidine, *N*-acetyl-1-methylhistidine were all reduced in OVER offspring compared with CON at day 90 of gestation (*p* < 0.02) while 1-methylhistamine was decreased at birth (*p* = 0.03). Branched chain amino acid (BCAA) metabolites, beta-hydroxyisovalerate, 3-methylglutaconate, and 3-hydroxyisobutyrate, were increased at day 90 and day 135 of gestation in OVER compared with CON (*p* < 0.02). Beta-hydroxyisovaleroylcarnitine was increased at day 90 (*p* < 0.02) but not day 135 or birth in OVER compared with CON. However, 4-methyl-2-oxopentanoate was decreased at day 90 of gestation in OVER compared with CON (*p* = 0.04). Metabolites involved in methionine, cysteine, and taurine metabolism were also increased in OVER compared with CON (*p* < 0.05). Specifically, *S*-methylcysteine was increased at all three time points in OVER compared with CON (*p* < 0.01). Taurine and *S*-methylcysteine sulfoxide were increased at day 135 (*p* < 0.02). Methionine sulfone was increased at birth (*p* = 0.05), and *S*-methylmethionine was increased at day 90 and birth (*p* < 0.02), but not day 135 compared with CON. In contrast, *N*-acetylmethionine was decreased in OVER at birth compared with CON (*p* < 0.01). Offspring of over-fed ewes also demonstrated alterations to glutathione metabolism. Specifically, GSH was increased at day 90 of gestation (*p* < 0.01) with no difference in oxidized glutathione. However, at day 90, cysteine-glutathione disulfide and 2-aminobutyrate were decreased in OVER compared with CON (*p* < 0.04). Ophthalmate was decreased at day 135 and *S*-methylglutathione was decreased at birth compared with CON (*p* < 0.05).

#### Maternal Over- and Restricted-Feeding Result in Different Metabolite Profiles in the Offspring

The different amino acid and lipid metabolite profiles in offspring as a result of restricted- or over-feeding in the dam are most notable when comparing the two groups directly with each other (Figure 2). In particular, RES offspring demonstrate increased 1-methylhistidine, 3-methylhistidine, *N*-acetyl-1-methylhistidine, and anserine at day 90 and day 135 compared with OVER offspring (*p* < 0.02), yet decreased imidazole propionate and imidazole lactate at all three time points compared with OVER offspring (*p* < 0.05; Figure 2). Branched chain amino acid metabolites were decreased overall in RES compared with OVER. Specifically, beta-hydroxyisovalerate, 3-methylglutaconate, and 3-hydroxyisobutyrate were decreased at day 90 and day 135 in RES compared with OVER (*p* < 0.02). In contrast, beta-hydroxyisovaleroylcarnitine was decreased at day 90 (*p* < 0.01) but increased at birth in RES compared with OVER (*p* < 0.01). Methionine, cysteine, and taurine metabolites were also altered in RES compared with OVER offspring. For example, S-methylcysteine was decreased in RES compared with OVER at all three time points (*p* < 0.01). Both reduced and oxidized glutathione were decreased at day 135 in RES compared with CON (*p* < 0.05). In contrast, *S*-methylglutathione was increased at birth (*p* < 0.02), 2-aminobutyrate was increased at day 135 (*p* < 0.01), and ophthalmate was increased at day 90 and day 135 in RES compared with OVER (*p* < 0.02).

Restricted maternal nutrition during gestation increased abundance of 118 of 268 (44%) identified lipid metabolites in RES compared with OVER (Figure 2). In particular, abundance of the polyunsaturated fatty acids eicosapentaenoate (EPA) and docosapentaenoate (n3 DPA) were increased in RES compared with OVER at all three time points (*p* < 0.02). Further, 10 fatty acid metabolites involved in acyl carnitine metabolism were increased in RES offspring compared with OVER at birth (*p* < 0.05). The ketone body 3-hydroxybutyrate was decreased at day 90 and increased at day 135 in RES offspring compared with CON (*p* < 0.04). Seventeen sphingolipid metabolites were reduced in RES compared with OVER at day 90 of gestation; however, sphingomyelin (d18:2/24:2) was increased compared with OVER at all three time points (*p* ≤ 0.02). Ceramide metabolites were also reduced at day 90 of gestation in RES offspring compared with OVER; however, glycosyl-*N*-stearoyl-sphingosine (d18:1/18:0) was increased in RES offspring compared with OVER at birth (*p* = 0.03).

## Discussion

**FIGURE 3:**
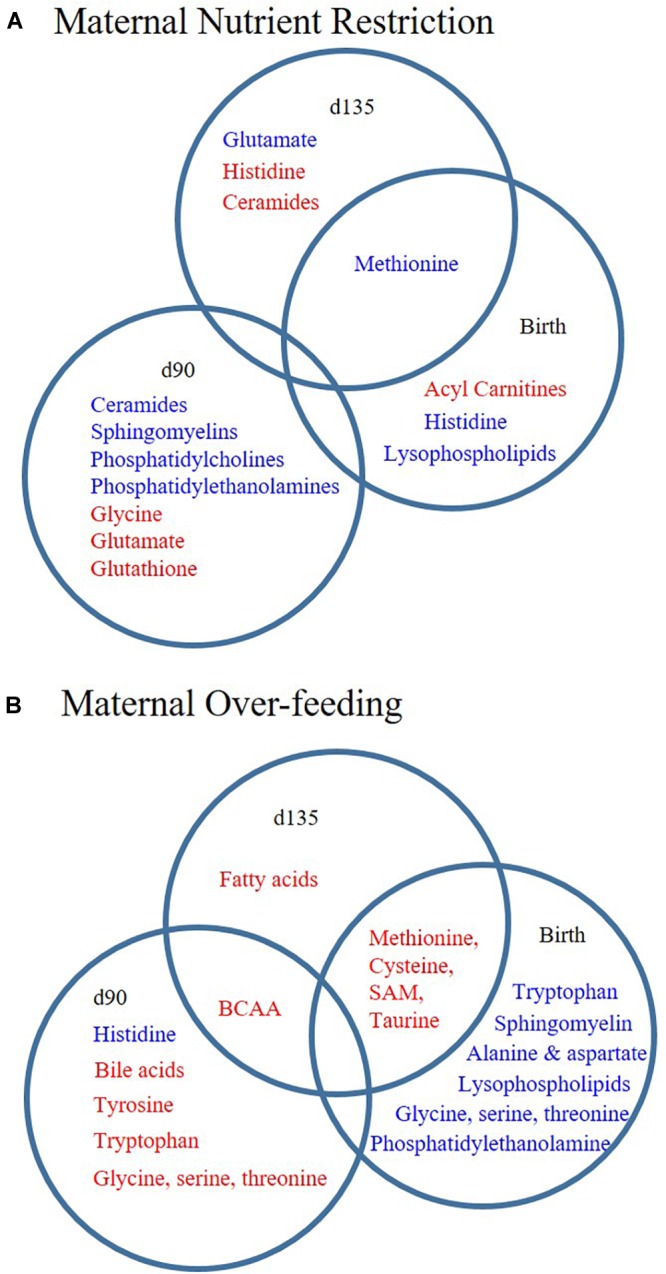
Poor maternal diet results in distinct lipid and amino acid metabolite profiles in offspring longissimus. Offspring from ewes fed a control (CON; 100% NRC), over-fed (OVER; 140% NRC), or restricted-fed (RES; 60% NRC) diet were sampled at days 90 or 135 of gestation, or within 24 h of birth. Metabolite sub-pathways increased (red) or decreased (blue) in abundance at each time point in RES vs. CON **(A)** or OVER vs. CON **(B)**. BCAA, branched chain amino acid; SAM, *S*-adenosyl methionine.

Fetal programming of muscle metabolism has potential consequences for post-natal growth and risk of several metabolic diseases in adulthood. Here, we investigated the effects of maternal diet on offspring muscle metabolism using two approaches. First, we assessed myoblast function in the presence of serum from RES and OVER offspring, allowing us to investigate if systemic factors in these offspring have an inhibitory effect on myoblast metabolism using a standard cell line. Second, we performed untargeted metabolomics on offspring LM to identify metabolic pathways impacted by maternal diet. Using these approaches, we found that (1) serum from RES offspring increased myoblast proton leak, indicating that maternal nutrient restriction changes circulating serum factors that result in redox imbalance of myoblasts; and (2) maternal over- and restricted-nutrition altered the offspring lipid and amino acid metabolite profile, but result in distinct metabolite profiles in offspring muscle (Figure 3).

Proton leak is associated with energy efficiency in the mitochondria, with increased leak correlated with reduced mitochondrial coupling and therefore, reduced efficiency in generating ATP (Rolfe et al., 1999). Proton pumping across the mitochondrial membrane and proton leak contribute to a cycle that dissipates redox energy (Rolfe et al., 1999). Additionally, the mitochondrial proton cycle contributes approximately 15% of the standard metabolic rate in skeletal muscle (Rolfe et al., 1999). Increased myoblast proton leak from RES serum could result in increased basal metabolic rate, thereby decreasing energetic efficiency. This may be a result of factors in the serum which increase oxidative stress and/or the inflammatory response in the muscle, altering mitochondrial function and/or efficiency. The serum contains a rich milieu of factors which could result in this change, which is an area for future study. Further, increased proton leak is associated with redox imbalance, and has been linked to increased oxidative stress as well as increased inflammatory responses (Li et al., 2017), which can result in reduced muscle growth. The lack of change in myoblast differentiation in response to fetal serum was not surprising, given that there are no differences in muscle weight or muscle fiber cross-sectional area at birth in response to poor maternal diet in the same experimental cohort (Gauvin et al., unpublished). However, in a similar model, we have demonstrated that over- and restricted-feeding during gestation results in decreased post-natal muscle growth in the early post-natal period (Reed et al., 2014). The use of serum in this model allowed us to determine if factors in the serum influenced muscle growth and/or metabolism, or if the changes in muscle are due to other influences, such as programmed changes in the myogenic precursor cells (pre- or post-natally) or the local muscle environment.

Metabolome analysis demonstrated that both maternal diet and day of gestation drive changes in LM metabolite abundance, including changes to amino acid, lipid, carbohydrate, and energy pathways. Samples within each time point (day 90, day 135, and birth) exhibited an overall similar metabolite composition. These observations suggest, that at the time points evaluated that the stage of development is a stronger regulator of metabolism than maternal nutrition. This is not unexpected given the vast changes in fetal muscle metabolism during gestation (Battaglia and Meschia, 1978). The fetus relies primarily on carbohydrates and amino acids for energy, rather than lipids, during pre-natal development. These metabolites provide an energy source for oxidative metabolism, protein synthesis, and structural development that occurs in muscle throughout gestation (Battaglia and Meschia, 1978). While lipid oxidation for energy is limited relative to carbohydrate and amino acid utilization, lipid uptake into muscle is critical for establishing cellular membranes and maintaining cellular integrity. Birth represents a change in nutrient availability and energy substrate utilization (Girard, 1990; Hillman et al., 2012). At birth, the maternal glucose supply is removed, and the diet becomes richer in lipid. Therefore, changes to tissue utilization reflect the change in fuel availability to increase lipid oxidation post-natally (Girard, 1990; Hillman et al., 2012).

Offspring of restricted-fed ewes exhibited significant changes in the lipid metabolite profile, including changes in ceramide, sphingolipid, and phospholipid metabolites. Sphingolipids are an essential part of lipid membranes (including the mitochondrial membrane) and serve as second messengers for signal transduction in pathways affecting cell growth, differentiation, stress responses, and apoptosis (Merrill et al., 1997; Khavandgar and Murshed, 2015). Complex sphingolipids, such as sphingomyelins, are formed from ceramide in the Golgi complex by the action of sphingomyelin synthases (Kudo et al., 2008; Villani et al., 2008). Catabolism of sphingomyelin is catalyzed by sphingomyelinase and results in the generation of free phosphocholine and ceramide. The roles for ceramide and sphingomyelins in muscle are varied and not completely understood. Sphingolipid signaling (via production of sphingolipid-1-phosphate from sphingomyelin) mediates entry of satellite cells into the cell cycle from quiescence, supporting post-natal muscle growth and repair (Nagata et al., 2006). While reduced presence of sphingomyelins in developing muscle at day 90 suggests inhibited muscle development, we did not identify any difference in the number of Pax7-positive muscle progenitor cells or the muscle fiber CSA in the LM (Gauvin et al., unpublished), which suggests alternate roles for sphingomyelins at this time point. For example, the sphingomyelin pathway has been implicated in insulin resistance. At day 90 of gestation, the reduction in ceramide and sphingolipid abundance in RES offspring compared with CON may indicate altered insulin dynamics in an attempt to adapt to restricted nutrient availability. Although these changes were not observed at birth, we and others have demonstrated altered insulin sensitivity in offspring during postnatal growth suggesting that early alterations to sphingomyelin metabolites may be a mechanism that programs offspring muscle for altered insulin sensitivity later in life.

Potential changes in insulin dynamics during late gestation and early post-natal life are supported by the increases in 3-hydroxybutrylcarnitine and acetyl carnitine at birth. In rats, 3-hydroxybutryl-carnitine is increased during periods of insulin resistance induced by fasting and high fat diet feeding (An et al., 2004). 3-hydroxybutyrl-carnitine was also increased during fasting-induced ketosis in adult males and in patients with type 2 diabetes (Soeters et al., 2012; Mai et al., 2013). The total amount of 3-hydroxybutyrl-carnitine correlated with the 3-hydroxybutyrate rate of appearance, suggesting that 3-hydroxybutyrl-carnitine concentration is related to 3-hydroxybutyrate flux (Soeters et al., 2012). Indeed, 3-hydroxybutyrl-carnitine is formed from 3-hydroxybutyrate in a coenzyme A and ATP dependent manner. Increased concentration of 3-hydroxybutyrl-carnitine may reflect a limitation of ketone body oxidation or may be a mechanism to prevent mitochondrial accumulation or prevent ketoacidosis (Boudin et al., 1976; Ramsay et al., 2001). Further, acetyl carnitine concentration was increased in patients with impaired glucose tolerance or type 2 diabetes and was positively correlated with increased body fat and waist to hip ratio (Mai et al., 2013; Abu Bakar and Sarmidi, 2017). Insulin dependent uptake of 2-deoxyglucose in cultured myotubes was inhibited by the acetyl carnitine in a dose dependent manner, suggesting that acetyl carnitine contributes to insulin resistance in skeletal muscle (Miyamoto et al., 2016). Further, in myotubes with increased mitochondrial dysfunction (induced by Antimycin A), acyl carnitine metabolites were increased, potentially due to incomplete beta oxidation by the mitochondria (Abu Bakar and Sarmidi, 2017). Together, these data suggest that the fetus attempts to adapt to the restricted nutrient environment by altering insulin sensitivity during mid-gestation.

Offspring of over-fed dams demonstrated significant changes in amino acid metabolism. Muscle is the primary storage site for amino acids in the body, and protein accretion during late gestation and post-natal growth is the primary mechanism for muscle hypertrophy. In the fetus, amino acids serve as a source of energy but also as building blocks for protein accretion. The amino acids present in the muscle depend on the provision of amino acids via circulation as well as the balance of *de novo* synthesis and catabolism of amino acids in the muscle itself. During periods of fetal stress, protein catabolism increases in the hind limb muscle, mainly due to increased metabolism of BCAA (Liechty and Lemons, 1984; Liechty et al., 1987). Amino acid supplementation to fetuses in late gestation increased leucine oxidation but did not increase muscle protein accretion in control offspring (Brown et al., 2012). In humans, maternal BMI was positively associated with BCAA abundance and their metabolic byproducts, which are associated with the development of obesity and insulin resistance (Newgard et al., 2009; Wang et al., 2011). Leucine is metabolized to generate alanine and glutamine. Alanine concentrations were increased at day 90 of gestation in offspring of over-fed dams, consistent with increases in BCAA metabolites reflecting leucine degradation (e.g., beta-hydroxyisovalerate and 3-methylglutaconate), some of which remained increased at day 135 of gestation. Further, increased abundance of 3-hydroxyisobutyrate as a result of BCAA catabolism is associated with fatty acid uptake and lipid accumulation in muscle, leading to insulin resistance in mice (Jang et al., 2016). Thus, alterations in BCAA may predispose offspring of over-fed dams to obesity and metabolic dysregulation at later stages of development.

The complex interplay between metabolism and epigenetics is just now beginning to be understood (reviewed in Etchegaray and Mostoslavsky, 2016); however, fluctuations in metabolite abundance are associated with alterations in histone methylation and acetylation. Decreased abundance of N6-acetyllysine in offspring of over-fed ewes at birth suggests the occurrence of epigenetic changes in the muscle as a result of poor maternal diet. Acetylation of lysine on specific residues in the N-terminal domains of histones is recognized as a marker of active gene transcription (Crane-Robinson et al., 1997). Decreased abundance of N6-acetyllysine suggest a closed chromatin structure and decreased gene transcription in the offspring of over-fed dams compared with controls. Acetylmethionine abundance was also decreased at birth, further suggesting altered epigenetic activity, as methionine is the penultimate methyl group donor (Chen and Riggs, 2011). Further, the availability of flavin adenine dinucleotide (FAD) in adipocytes regulates the methylation status (and therefore transcription) of genes associated with energy expenditure such as PGC-1α and the pyruvate dehydrogenase kinase 4 (PDK4) through the activity of lysine-specific demethylase-1 (LSD1; Hino et al., 2012). In our data, at day 90 of gestation, offspring of over-fed dams demonstrated increased concentrations of FAD, suggesting altered methylation status of LSD1 target genes and possibly other epigenetic modifications.

Additionally, at day 90 of gestation, glucose-6-phosphate (G-6-P) and Coenzyme A were increased coupled with reduced adenosine and adenosine monophosphate. The increased G-6-P may contribute to the increased GSH at this time point through the pentose phosphate pathway, which uses G-6-P to generate NADPH via glucose-6-phosphate dehydrogenase (Diaz-Flores et al., 2006). Glutathione reductase then uses NADPH as an electron donor cofactor to generate reduced glutathione (GSH). This may be a protective mechanism against oxidative stress as a result of maternal over-nutrition. Indeed, GSH was increased at day 90 of gestation in response to increased maternal nutrient intake. Together, these data suggest that over-feeding during gestation alters muscle protein metabolism in the offspring in ways that likely predispose the offspring for reduced protein synthesis, increased oxidative stress, and altered post-natal muscle metabolism. Importantly, these changes may be programmed by epigenetic mechanisms, resulting in long-lasting effects that may span multiple generations.

As expected, the metabolite profiles of offspring of restricted- and over-fed dams were divergent in lipid and amino acid metabolism. For example, BCAA metabolites were reduced at day 90 of gestation and increased at birth in RES offspring compared with OVER offspring. Acyl carnitine moieties were increased in RES compared with OVER offspring at birth, suggesting divergent fatty acid metabolism. Further, sphingomyelins and ceramides were decreased in RES compared with OVER, suggesting alterations in fatty acid metabolism as well as insulin sensitivity. These data highlight the vast differences in the metabolic response of the muscle, despite similar phenotypes observed in offspring of poorly nourished ewes (Reed et al., 2014). Importantly, this demonstrates that despite similar phenotypic outcomes, over- and restricted-feeding during gestation result in distinct muscle metabolite profiles.

## Conclusion

Poor maternal nutrition during gestation can negatively affect muscle and offspring development. We demonstrate here that over- and restricted-feeding during gestation alters metabolite abundance in the offspring muscle in a diet-specific manner. Together, these data suggest that poor maternal diet during gestation causes metabolic changes in offspring skeletal muscle, which likely have long-lasting effects on the potential for muscle growth, muscle metabolism, and importantly, whole body metabolism and energy efficiency.

## Ethics Statement

This study was carried out in accordance with the recommendations of the University of Connecticut Institutional Animal Care and Use Committee. The protocol was approved by the University of Connecticut Institutional Animal Care and Use Committee.

## Author Contributions

DM participated in design and coordination, analyzed data, and drafted the manuscript. AJ, SP, MH, KM, KG, and SZ participated in design and coordination and critically evaluated the manuscript. SR conceived of the study, participated in design and coordination, analyzed data, and drafted the manuscript. All authors read and approved the final manuscript.

## Conflict of Interest Statement

The authors declare that the research was conducted in the absence of any commercial or financial relationships that could be construed as a potential conflict of interest.

## Footnotes

1 https://software.broadinstitute.org/morpheus
